# Analysis of reasons for screen failure by participant race in the Phase 2 Auτonomy study in early Alzheimer's disease

**DOI:** 10.1002/alz70859_100107

**Published:** 2025-12-25

**Authors:** Martine Meyer, David Henley, Jennifer Bogert, Maggie Fedgchin, Kimberly Fisher, Gallen Triana‐Baltzer, Ziad S. Saad, Pieter‐jan Berghmans, Janice Wong

**Affiliations:** ^1^ Johnson & Johnson, San Diego, CA USA; ^2^ Johnson & Johnson, Titusville, NJ USA; ^3^ Johnson & Johnson, Raritan, NJ USA; ^4^ Neuroscience Biomarkers, Johnson & Johnson Innovative Medicine, La Jolla, CA USA; ^5^ Johnson & Johnson, Beerse, Antwerpen Belgium; ^6^ Johnson & Johnson, Cambridge, MA USA

## Abstract

**Background:**

An important goal in Alzheimer's disease (AD) clinical trials is the recruitment of a cohort that represents the ethnic/racial diversity of the general patient population. We report preliminary data on the rates and reasons for screen failure (SF) across races in the ongoing Phase 2b Auτonomy (NCT04619420) study of posdinemab (JNJ‐63733657), an anti‐phosphorylated‐tau (anti‐P‐tau) monoclonal antibody, in participants with early symptomatic AD.

**Method:**

SF participants were identified from the Auτonomy study. Race of participants was categorized as White, Black/African American, Asian, American Indian/Alaska Native, and Native Hawaiian/Other Pacific Islander. SF rates and reasons (categorized according to study inclusion and exclusion criteria) in each race were evaluated.

**Result:**

Of 2563 participants screened, 2040 failed to meet eligibility criteria for study participation, for an overall SF rate of 79.6%. SF rates were numerically highest in the Black/African American population (86.4% [51/59]), followed by White (80.5% [1690/2100]), and Asian (73.3% [206/281]) populations. The most common reason for SF amongst these three races was not meeting the inclusion criterion for evidence of pathologically elevated plasma p217tau concentration (Black/African American: 35.3% [18/51], White: 38.6% [652/1690], and Asian: 26.7% [55/206]). The second most common reason was not meeting the early AD clinical inclusion criterion (defined as gradual progressive subjective cognitive decline over past ≥6 months, Clinical Dementia Rating‐Global Score = 0.5 and memory box score ≥0.5); SF due to not meeting the early AD inclusion criterion was numerically higher for the Black/African American population (21.6 % [11/51]), compared with White (12.1% [204/1690]) and Asian (18.0% [37/206]) populations. Other reasons for SF will be presented.

**Conclusion:**

This analysis showed numerically higher overall SF rates amongst Black/African American population compared with other races evaluated. The Black/African American population had a higher proportion of SF due to the early AD clinical inclusion criterion compared with the White population; however, comparison is limited by the low number of Black/African American participants screened. Analysis of SF data from existing AD trials may help inform on recruiting a diverse study population by furthering understanding of any racial differences in underlying AD pathophysiology levels or co‐morbidities contributing to cognitive impairment.

